# A nano-enhanced vaccine for metastatic melanoma immunotherapy

**DOI:** 10.20517/cdr.2021.132

**Published:** 2022-07-07

**Authors:** Katelyn E. Salotto, Walter C. Olson Jr, Karlyn E. Pollack, Anuradha Illendula, Elishama Michel, Sydney Henriques, Todd Fox, Susan Walker, Marya Dunlap-Brown, Craig L. Slingluff Jr, Mark Kester, Helena W. Snyder

**Affiliations:** ^1^School of Engineering and Applied Sciences, University of Virginia, Virginia, VA 22904, USA.; ^2^Department of Surgery, School of Medicine, University of Virginia, Virginia, VA 22903, USA.; ^3^Department of Dermatology, School of Medicine, University of Virginia, Virginia, VA 22903, USA.; ^4^Department of Pharmacology, School of Medicine, University of Virginia, Virginia, VA 22903, USA.; ^5^University of Virginia nanoSTAR Institute, Virginia, VA 22903, USA.; ^6^Molecular Immunologic & Translational Sciences (MITS) Core, School of Medicine, University of Virginia, Virginia, VA 22903, USA.; ^7^Department of Materials Science, School of Engineering and Applied Sciences, University of Virginia, Virginia, VA 22903, USA.

**Keywords:** Nanoliposomes, nanoscale drug delivery, cancer vaccines, metastasized melanoma, peptides, melanoma drug resistance

## Abstract

**Aim**: Despite the huge advancements in cancer therapies and treatments over the past decade, most patients with metastasized melanoma still die from the disease. This poor prognosis largely results from resistance to conventional chemotherapies and other cytotoxic drugs. We have previously identified 6 antigenic peptides derived from melanomas that have proven efficacious for activating CD4^+^ T cells in clinical trials for melanoma. Our aim was to improve pharmacodynamics, pharmacokinetic and toxicological parameters by individually encapsulating each of the 6 melanoma helper peptides within their own immunogenic nanoliposomes.

**Methods**: We modified these liposomes as necessary to account for differences in the peptides’ chemical properties, resulting in 3 distinct formulations. To further enhance immunogenicity, we also incorporated KDO2, a TLR4 agonist, into the lipid bilayer of all nanoliposome formulations. We then conducted *in vivo* imaging studies in mice and *ex vivo *cell studies from 2 patient samples who both strongly expressed one of the identified peptides.

**Results**: We demonstrate that these liposomes, loaded with the different melanoma helper peptides, can be readily mixed together and simultaneously delivered without toxicity *in vivo*. These liposomes are capable of being diffused to the secondary lymphoid organs very quickly and for at least 6 days. In addition, we show that these immunogenic liposomes enhance immune responses to specific peptides *ex vivo*.

**Conclusion**: Lipid-based delivery systems, including nanoliposomes and lipid nanoparticles, have now been validated for pharmacological (small molecules, bioactive lipids) and molecular (mRNA, siRNA) therapeutic approaches. However, the utility of these formulations as cancer vaccines, delivering antigenic peptides, has not yet achieved the same degree of commercial success. Here, we describe the novel and successful development of a nanoliposome-based cancer vaccine for melanoma. These vaccines help to circumvent drug resistance by increasing a patient’s T cell response, making them more susceptible to checkpoint blockade therapy.

## INTRODUCTION

Melanoma of the skin is the 5th most common cancer for both males and females in the United States.^[1]^, and currently, only complete surgical removal is considered curative. In advanced malignant and metastasized stages of melanoma, where surgery alone cannot offer remission, the prognosis is extremely poor. This is because human melanoma is particularly resistant to conventional chemotherapies and other cytotoxic drugs^[2-4]^, and this resistance has primarily been linked to dysregulations in apoptosis^[2]^.

Recent progress in cancer immunotherapy has prolonged median survival for metastatic melanoma from about nine months to about 3 years, but most patients with metastatic melanoma still do not survive^[5-7]^. A lack of pre-existing T cell immunity to cancer antigens is commonly a root cause of failure. In addition, both intrinsic and adaptive resistance to immunotherapies in melanoma has been observed^[4]^. Therefore, there is a significant need for new therapies to prevent melanoma recurrence by enhancing immune responses to melanoma. Cancer vaccines offer the promise to induce immune responses to cancer when spontaneous antitumor immunity is absent or weak. The effectiveness of immune checkpoint blockade therapy demonstrates that melanoma cells express antigens that can be recognized by CD4 and CD8 T cells, and also that T cell responses to those antigens can mediate tumor regression, when tumor-associated immune dysfunction is abrogated. There has been recent enthusiasm for approaches targeting antigens created by spontaneous mutations (mutated neoantigens)^[8]^; however, recent data highlight the value of shared melanocytic antigens as relevant targets^[9]^, and other data highlight the value of vaccinating against shared cancer-testis antigens^[10]^.

Data from our group and others have highlighted the pivotal role of CD4^+^ (helper) T cells in cancer immunity and cancer control^[11-14]^. We have previously developed a multi-peptide melanoma vaccine consisting of 6 melanoma helper peptides (6MHP) designed to induce melanoma-reactive CD4^+^ T cells, and found that they are immunogenic in humans, inducing objective clinical responses and very high rates of 5-year survival. Additionally, we found that the immune responses are enhanced when simultaneously injecting a toll-like receptor 3 (TLR3) agonist with the vaccine^[15]^. With a different peptide vaccine, we also found that agonists for TLR4 are useful vaccine adjuvants alone or with incomplete Freund’s adjuvant^[16]^. However, these responses are still modest in absolute magnitude, even with strong adjuvants, and even the best responses usually represent less than 1% of circulating CD4^+^ T cells. A major goal of current cancer vaccines is to induce stronger and more durable T cell responses, but optimal vaccine strategies in humans have not yet been defined.

Most cancer vaccines contain antigens plus vaccine adjuvants. However, when they are co-administered as soluble agents, there is no assurance that the antigens and the adjuvants will be delivered to the same antigen-presenting cell. Nanoliposomes provide a way to deliver antigens to phagocytic antigen-presenting cells, especially dendritic cells, while simultaneously delivering vaccine adjuvants to the same antigen-presenting cells. The use of nanoliposomes to deliver cancer therapeutics offers significant promise and has had several clinical successes^[17]^. Indeed, many vaccines benefit from lipid-based delivery. Of particular note are the recently authorized COVID-19 vaccines, which utilize lipid nanoparticles containing antigen-encoding RNA^[18,19]^. Our team has been successful in the use of nanotechnologies, including nanoliposomes, for a host of different therapies^[20-25]^. Nanoliposomes have the capability of altering the pharmacokinetic properties of antigens and immunomodulators/adjuvants, with a marked reduction in off-target toxicity, protection of the therapeutic from degradation, along with more specific targeting to antigen-presenting cells^[26]^. A theoretical benefit of using a nanoliposome vaccine over merely injecting antigens and adjuvants individually is that the nanoliposome ensures all adjuvants circulate together and are delivered to the antigen-presenting cells at the same time. Furthermore, nanoliposomes are readily taken up by the antigen-presenting dendritic cells (DCs)^[27-30]^; therefore, having a TLR agonist incorporated into the liposome should provide increased CD4^+^ T cell activation. Taken together, nanoliposomes are ideal nano-carriers and delivery agents for emerging cancer vaccines due to their versatility, ease of modification, and ability to deliver several adjuvants simultaneously^[31]^.

As described above, one of the advantages of liposomal delivery is the ability to incorporate vaccine adjuvants into the peptide delivery vehicle. We have chosen to use the toll-like-receptor 4 (TLR4) agonist, KDO2-lipid A (herein KDO2, but also often referred to as KLA). KDO2 is a synthetic and homogenous form of Lipid A, an essential component of lipopolysaccharides (LPS) in Gram-negative bacteria^[32]^. KDO2 stimulates potent and reproducible host immune responses through the complex of TLR4 and myeloid differentiation protein 2^[32-34]^. In previous studies, we have used naturally-derived LPS as a TLR4 agonist^[16]^, and others have used KDO2 and other lipid A derivatives in several cancer immunotherapies^[35-38]^; thus, it is an appropriate choice of agonist for this study. Here, we want to improve pharmacodynamics, pharmacokinetic and toxicological profiles by incorporating KDO2 directly into the delivery system to enhance CD4^+^ T cells via TLR4. Thus, we hypothesized that (1) we could develop stable KDO2-containing nanoliposomes for the encapsulation of each of the 6 melanoma helper peptides identified previously by our team; and (2) these nanoliposomes would trigger an increased CD4^+^ T-cell response over and above delivery of the peptide(s) or TLR4 agonist alone.

## METHODS

### Encapsulation of melanoma peptides within nanoliposomes

The 6 melanoma peptides that induce melanoma-reactive CD4^+^ T cells in patients, as well as their protein epitope and ideal pH range for solubility, are provided in Table 1. We initially chose a neutral formulation to encapsulate all peptides and then, through an iterative process, modified formulations to optimize encapsulation efficiencies for several of the peptides. Through this process, we identified 3 different nanoliposome formulations (neutral, cationic and anionic) suitable for distinct peptides. However, all of our formulations contained the same base lipid components, including 1,2-distearoyl-sn-glycero-3-phosphocholine (DSPC); 1,2-dioleoyl-sn-glycero-3-phosphoethanolamine (DOPE); 1,2-distearoyl-sn-glycero-3-phosphoethanolamine-N-[methoxy(polyethylene glycol)-2000] (ammonium salt) (PEG2000PE); KDO2; cholesterol; and a fluorophore [either rhodamine or 1,1'-dioctadecyl-3,3,3',3'-tetramethylindodicarbocyanine, 4-chlorobenzenesulfonate salt (DiD)]. Each formulation contained DSPC and DOPE at a 2.14:1 molar ratio to form a stable, spherical nanoparticle. In addition, cholesterol at a 30 molar percent was incorporated to increase rigidity and reduce leakiness. PEG(2000)-PE at 2.5 molar percent was incorporated for biological stability, KDO2 was added to all formulations at 0.1 molar percent, and a fluorescent probe (rhodamine or DiD) was added at 0.2 molar percent for subsequent biodistribution imaging studies. Keeping the molar ratios of these key components constant, we incorporated hexadecyl phosphate (DHP) at 10 molar percent for the anionic formulation and positively charged lipid 1,2-dioleoyl-3-trimethylammonium-propane (chloride salt) (DOTAP) at 7 molar percent for the cationic formulation. All lipid components were dissolved in chloroform and mixed in the ratios shown in table 2. The lipid mixtures were then dried down in a nitrogen blower for roughly 2 h until all of the chloroform was evaporated. Then, the peptide solutions (or 1× PBS for the “ghost” formulation) were added to the dried down lipids; the tubes were then vortexed and placed in a heat shaker at 60 °C for 2 h, followed by sonication in a 60 °C sonic bath for roughly five minutes. After sonication, the liposomes were extruded through a 100-nanometer pore membrane eleven times to create uniformly sized liposomes. The extruded liposomes were subsequently run through a Sepharose gel bead column to separate the liposomal drugs from the free drug. The solution was analyzed using dynamic light scattering (DLS) (polydispersity, an average hydrodynamic diameter of particles in solution) and mass spectrometry.

**Table 1:** Amino acid sequence of the 6 melanoma helper peptides along with their abbreviation, Epitope (the sequence of amino acids recognized by the T cell receptor, which defines the specificity of the response) and ideal pH range required for stability and dissolution

**Amino acid sequence** **(letter = 1 amino acid)**	**Abbreviation**	**Epitope** **(protein, residue numbers)**	**Ideal pH range**
AQNILLSNAPLGPQFP	AQN	Tyrosinase_ 56-70_	8.5-9.0
WNRQLYPEWTEAQRLD	WNR	gp100 _44-59_	7.0-8.0
LLKYRAREPVTKAE	LLK	MAGE-1,2,3,6_ 121-134_	6.0-8.0
FLLHHAFVDSIFEQWLQRHRP	FLL	Tyrosinase _386-406_	6.0-8.0
RNGYRALMDKSLHVGTQCALTRR	RNG	Melan-A/MART-1 _51-73_	6.0-8.0
TSYVKVLHHMVKISG	TSY	MAGE-3 _281-295_	5.0-5.5

To further ensure optimal encapsulation efficiency of the MHPs, we utilized distinct dissolution buffers tailored to each peptide’s optimal pH range and previously optimized for *in vivo* injection of the free peptides into patients enrolled in our earlier clinical studies. Specifically, for the neutral peptide formulations, 1.33 mg/mL sodium bicarbonate (NaHCO_3_) in a 1:2 solution of lactated Ringer’s solution (LR) and water, respectively (B1); for the cationic formulation, 5 mg/mL NaHCO_3_ in water (B2); for the WNR anionic formulation, 1 mg/mL NaHCO_3_ in water (B3); and for the TSYVKVLHHMVKISG (TSY) anionic formulation, 1:9 ratio of 2-(N-morpholino) methanesulfonic acid (MES) buffer and water (B4).

Liposomal stability and peptide release studies were conducted by storing the liposomes in different conditions and media. We monitored the liposomes over a 5-week period in both 1X PBS and 10% fetal bovine serum, under refrigerated, room temperature, and body temperature storage conditions. We ran DLS studies, as well as centrifugation followed by mass spectrometry of the supernatant and reconstituted liposome solutions to monitor liposome stability and peptide release, respectively.

### *In vivo* murine studies

A 6-day study was performed to determine the biodistribution of the fluorescent nanoliposomes, utilizing daily live-mouse imaging with an *in vivo* imaging system (IVIS). For this study, four mice received a DiD fluorescently labeled 6MHP-KDO2-nanoliposome injection. Two mice were injected subcutaneously (SQ) into either flank and 2 mice were injected intravenously (IV). Each peptide nanoliposome had been previously formulated and stored separately. On the day of the experiment, all 6 formulations were mixed together in ratios that contained 0.4 μg of each peptide. The mice were administered isoflurane anesthesia. On day zero, once asleep, the mice were injected with the 6MHP-KDO2-nanoliposome mixture and then transferred immediately to the IVIS instrument. Imaging was conducted within 30 s (or less) of injection. For IVIS® Spectrum Image collection and processing, tomographic fluorescent images were collected of all mice using epi-illumination on a Caliper IVIS® Spectrum scanner following anesthetization with inhaled isoflurane inside a conduction chamber. Live imaging was then performed daily for 6 days. On the 6th day, the mice were euthanized, and their organs were harvested for follow-up *ex vivo *studies. The organs selected for harvest included the lungs, liver, spleen, kidneys, and lymph nodes. Organs from the *in vivo *study were transferred to 24-well plates, and fluorescent imaging data were collected on the IVIS® Spectrum Image system for qualitative determination of biodistribution. Fluorescent image collection used Living Image® software by Caliper Life Sciences. Image processing was done by importing Living Image® files into Aura imaging software by spectral instruments imaging, and then performing region of interest (ROI) measurements to quantify the fluorescence emitted from the mice. The fluorescent light images were collected in the same manner for the harvested organs following the live animal imaging once the mice were sacrificed. The measurement for fluorescence is the mean radiant efficiency within the drawn ROI. Radiant efficiency describes the fluorescent energy emitted from the specimen as a fraction of the excitation fluorescent radiation released by the scanner and incident upon the specimen. Mean radiant efficiency is unitless and is used so that comparisons may be made among mice and harvested organs of different sizes, and consequently slightly differing areas within ROIs. ROIs were drawn as subject ROIs according to the Living Imaging® software user manual (Caliper Life Sciences, 2012).

### *Ex vivo* lymphocyte stimulation using immunogenic TSY encapsulated nanoliposomes

Freshly obtained (cryopreserved) peripheral blood mononuclear cells (PBMC) and lymphocytes from the sentinel immunized nodes (SIN) of 2 patient donors (under IRB protocols, clinical trial Mel41 (NCT00089219; IRB #10464)^[39]^ were utilized to assess CD4^+^ T cell proliferation after treatment with the anionic TSY (MAGE-3_281-295_ peptide; Table 1) MHP-KDO2 nanoliposome. Firstly, a cell viability study was performed using a live-dead marker and flow cytometry to ensure that cell viability was not impacted by the presence of either the peptides or the liposomes. In this study, SIN lymphocytes from the 2 patient donors (SIN1and SIN2) were treated with a variety of free peptides and liposome combinations. For the free peptide group, the treatments included: no peptide and GAG peptide as negative controls, a mixture of all 6MHPs, and the single TSY peptide of interest. For the liposome group, the cells were treated with ghost liposomes, liposomes containing KDO2 but no peptide, and TSY containing liposomes both with and without KDO2. A control cell line was used as a viability comparison. Next, in order to assess CD4 T cell proliferation, we split the SIN1 and SIN2 cell lines into five treatment groups. These were: free non-encapsulated TSY, a ghost nanoliposome, a ghost KDO2 nanoliposome, a TSY encapsulated (no KDO2) nanoliposome, and a combinatorial KDO2/TSY nanoliposome (full vaccine). A CFSE dye-dilution proliferation assay was performed to evaluate the donor immune response by flow cytometry. Specimens were thawed and labeled with carboxyfluorescein diacetate (CFSE; Vybrant® CFDA SE Cell Tracer Kit, Invitrogen™, ThermoFisher Scientific) according to the manufacturer’s instruction, with a final dye concentration of 1 µM. Two hundred thousand labeled cells were added to flat bottom wells of a 96-well cluster plate (Falcon, ThermoFisher) containing peptides in solution or in nanoparticle form. The final peptide concentration was 2 µg/mL in a final culture volume of 0.2 mL. We also utilized a media-only and an HIV GAG peptide as negative controls^[40]^. To avoid cell starvation from the high PBS content of the nanoliposomes’ solvent relative to the necessary culture media nutrient content to sustain the culture during incubation, cells and liposome-containing formulations of peptide (and liposome controls) were first incubated (“pulsed”) for 2 h at 37 °C followed by centrifugation to pellet the cells (and absorbed or internalized nanoparticles). The “pulsing” medium supernatant was removed and replaced with a complete culture medium consisting of AIM V (Gibco/Life Technologies, ThermoFisher) supplemented with 5% human AB serum (Gemini Bioproducts). Treatments were incubated for five days. At the end of incubation, cells were collected and labeled with a live/dead fixable dye (Aqua; ThermoFisher), followed by labeling with CD3 v450, CD4 PE, and CD8 PE-Cy7 (BDBiosciences). Cells were acquired on a Canto II flow cytometer (BDBiosciences) maintained by the Carter Immunology Center at the University of Virginia and the data were analyzed using FlowJo software (version 10; BDBiosciences).

## RESULTS

### Physico-chemical characterization of 6MHP nanoliposomal formulations

We initially incorporated each of the 6 melanoma helper peptides (6MHP) in a neutral liposome formulation containing: (DSPC); (DOPE); (PEG2000PE); KDO2; cholesterol; and a fluorophore in ratios of 4.60:2.14:0.25:0.01:3.00:0.02 respectively [Table 2]. After quantifying initial encapsulation masses, we utilized an iterative approach to improve encapsulation via modifying the charge and lipid ratios of the liposomal formulation as well as the buffer for the peptide. The proportional improvements in encapsulation, as well as the encapsulation efficiencies, are depicted in Table 3.

**Table 2:** Finalized liposome formulations for the neutral, cationic and anionic nanoliposomes developed to encapsulate different peptides based on their residual charge. A dissolution buffer maintaining each peptide’s optimal pH range was also used during fabrication, where B1= 1.33 mg/mL sodium bicarbonate (NaHCO_3_) in a 1:2 solution of Lactated Ringers solution (LR) and water respectively; B2 = 5 mg/mL NaHCO_3_ in water; B3 = 1 mg/mL NaHCO_3_ in water; and B4 = 1:9 ratio of 2-(N-morpholino) methanesulfonic acid (MES) buffer and water

**Lipid Component**	**Molar Ratio**		
	**Neutral (FFL,LLK,RNG)**	**Cationic (AQN)**	**Anionic (WNR, TSY)**
DSPC	4.60	4.12	3.91
DOPE	2.14	1.90	1.81
PEG(2000)-PE	0.25	0.25	0.25
KDO2	0.01	0.01	0.01
Cholesterol	3.00	3.00	3.00
Rhodamine (or DiD)	0.02	0.02	0.02
DOTAP	-	0.70	-
DHP	-	-	1.00
Buffer	B1	B2	B3 (WNR), B4 (TSY)

DSPC: 1,2-distearoyl-sn-glycero-3-phosphocholine; DOPE: 1,2-dioleoyl-sn-glycero-3-phosphoethanolamine; DOTAP: 1,2-dioleoyl-3-trimethylammonium-propane (chloride salt).

**Table 3:** Showing both the Initial peptide encapsulation mass within our neutral nanoliposome formulation when the peptides are dissolved in 1 × PBS; compared to the encapsulation efficiencies within our optimized nanoliposome formulations, where each peptide is also dissolved in an aqueous solution that maintains optimal pH, peptide stability, and dissolution. Encapsulation efficiency is based upon initial 500 μg/ml peptide concentration. All peptides were calculated from a full vaccine, which included all lipid components (i.e. adjuvents, PEG, peptides). Each optimized mass encapsulation is based on *n* = 3 separate experiments, repeated in triplicate

**Peptide**	**Original mass encapsulation using neutral formulation and 1X PBS** **(μg/mL)**	**New liposome formulation (Charge and buffer)**	**pH range**	**Optimized mass encapsulation** **(μg/mL)**	**Encapsulation improvement **	**Encapsulation efficiency (%)**
AQN	0.02 0.005	Cationic (B2)	8.5-9.0	6.25 1.13	312X	1.25
WNR	4.81 0.09	Anionic (B3)	7.0-8.0	7.64 0.99	1.59X	1.53
LLK	34.56 0.69	Neutral (B1)	6.0-8.0	57.26 14.39	1.66X	11.45
FLL	28.15 2.05	Neutral (B1)	6.0-8.0	58.61 4.96	2.08X	11.72
RNG	22.37 0.30	Neutral (B1)	6.0-8.0	19.18 6.36	0.85X	3.29
TSY	0.17 0.001	Anionic (B4)	5.0-5.5	141.35 2.18	831X	28.27

AQN: AQNILLSNAPLGPQFP; WNR: WNRQLYPEWTEAQRLD; LLK: LLKYRAREPVTKAE; FLL: FLLHHAFVDSIFEQWLQRHRP; RNG: RNGYRALMDKSLHVGTQCALTRR; TSY: TSYVKVLHHMVKISG.

**Figure 1:**
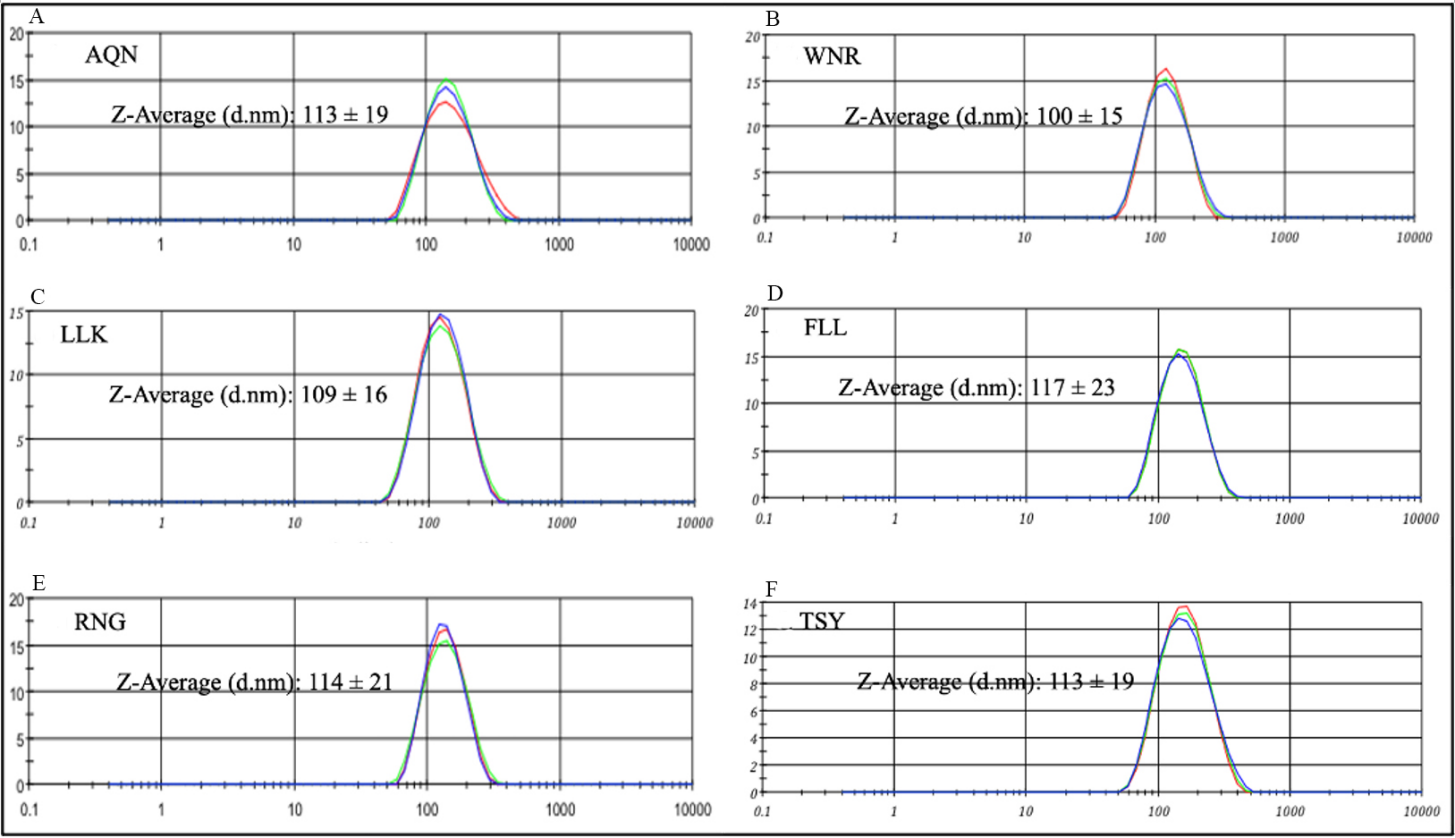
Typical dynamic light scattering (DLS) data attained for each of the 6 melanoma helper peptides. The data shows size distribution by intensity with size, d in nanometers along the X-axis and percentage intensity on the Y axis. (A) shows the DLS profile of the AQN peptide; (B) the DLS profile of the WNR peptide; (C) is that of LLK; (D) FLL; (E) RNG; and (F) is the typical profile for the TSY peptide. The graphs show a homogenous size distribution for all 6 peptides. The Z-average size (d.nm) attained from multiple samples is also provided for each peptide. AQN: AQNILLSNAPLGPQFP; WNR: WNRQLYPEWTEAQRLD; LLK: LLKYRAREPVTKAE; FLL: FLLHHAFVDSIFEQWLQRHRP; RNG: RNGYRALMDKSLHVGTQCALTRR; TSY: TSYVKVLHHMVKISG.

**Figure 2:**
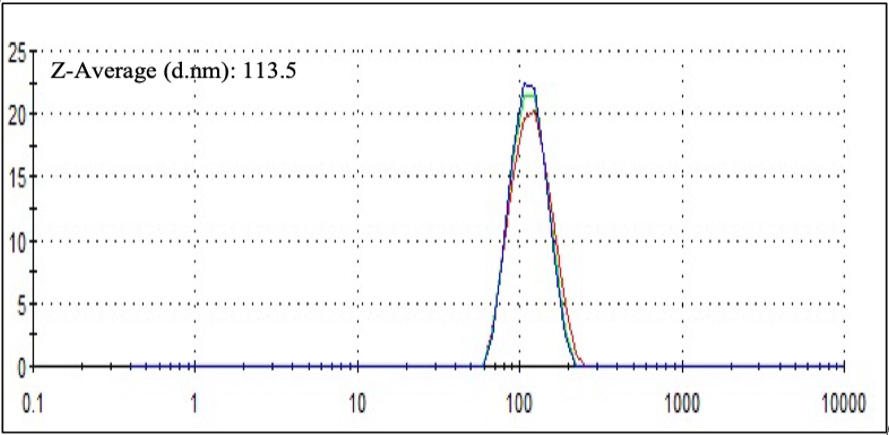
Dynamic light scattering data attained after mixing all 6 peptide nanoliposome formulations together. The data shows size distribution by intensity, with size (d) in nanometers along the X-axis and percentage intensity on the Y axis. The graphs show a homogenous size distribution. The Z-average size (d.nm) attained for this data is 113.5 nm.

Using dynamic light scattering (DLS, Malvern Instruments), we demonstrated that all 6 optimized formulations displayed stable, consistent, and homogeneous size distribution, with an overall average size range of 113 ± 8 nm when averaged among all 6 formulations [Figure 1]. Furthermore, Figure 2 shows that all 6 formulations could be mixed together and remain stable in suspension, providing confidence that the charged nanoliposomes are not interacting with each other to cause destabilization or aggregation. The mixed sample gave a Z-average size of 113.5 nm, which is in agreement with our average size distribution of the individual nanoliposomes.

In addition, results of a 5-week long release kinetics study and analysis by LC-MS did not detect any measurable amount of peptides present in the MHP-KDO2-nanoliposome supernatant when stored at 4 °C (data not shown). These data suggest that the nanoliposomes do not have a significant release of encapsulated peptides from their core for at least that length of time, while under typical refrigerated storage conditions. Additionally, the supernatant collected remained clear, providing confidence that the fluorophore was not released from the liposome and DLS analyses still showed good liposome stability. Conversely, at body temperature, greater than 50% of the peptide concentration was released within the first 24 h. DLS data showed that the liposome remained intact, even in 10% bovine serum and the supernatant remained clear of the fluorophore. After one week, the liposomes kept at room and body temperature did appear to begin breaking down, shown by an increased number of peaks in the DLS analyses (data also not shown).

### Murine biodistribution studies

**Figure 3:**
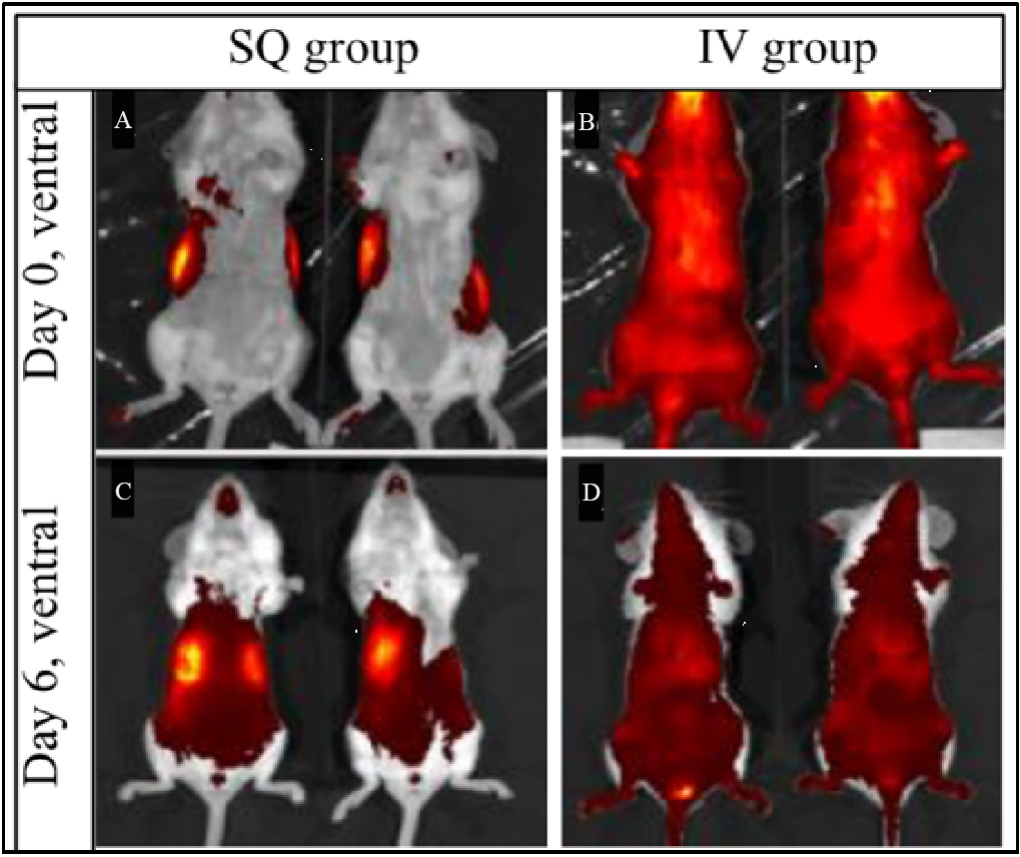
Fluorescent imaging of liposome biodistribution in mice on days 0 and 6. Two mice (SQ group) were injected subcutaneously in either flank and 2 mice (IV group) were injected intravenously.

**Figure 4:**
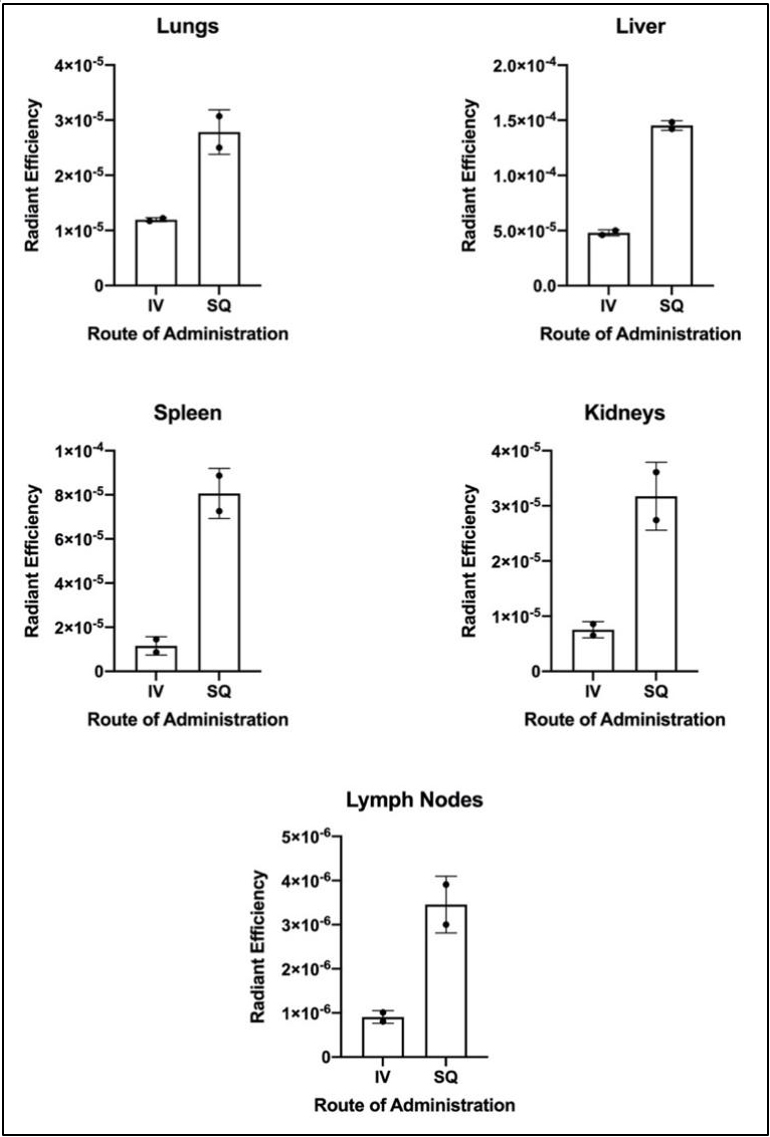
Showing the mean radiant efficiency in the extracted organs of four mice, 2 of which were injected with fluorescent and immunogenic liposomes via intravenous (IV) injection and 2, who were administered the same nanoliposomes via subcutaneous (SQ) injection in either flank.

A biodistribution study using a mixture of all 6 MHP formulations was performed using an IVIS Spectrum imaging system. Since each liposome formulation contained a different peptide mass, the mixture was prepared to contain equal amounts of each peptide (i.e., different volumes of each liposome solution to maintain equal peptide delivery). For this particular study, the nanoliposome vaccine was prepared using (DiD) fluorophore, as DiD has a far-infrared emission spectrum conducive to live animal imaging via IVIS. We compared routes of administration, SQ vs. IV. The IVIS images on day zero (immediately after injection) and day 6 (6 days after injection) are shown in Figure 3. The mice injected SQ displayed little bio-distribution on day zero [Figure 3A], which intensified within a region consistent with liver and spleen on day 6 [Figure 3C]. In contrast, images of the mice injected IV with nanoliposome vaccines show rapid, systemic biodistribution in both mice on day zero [Figure 3B], but the tissue fluorescence diminished by day 6 [Figure 3D] as compared to the fluorescence on day zero. On day 6, the mice were sacrificed and their organs were harvested. Quantitative fluorescence measurements were performed using Aura region of interest (ROI) programming and software (see methods), and these results are shown in Figure 4.

**Figure 5:**
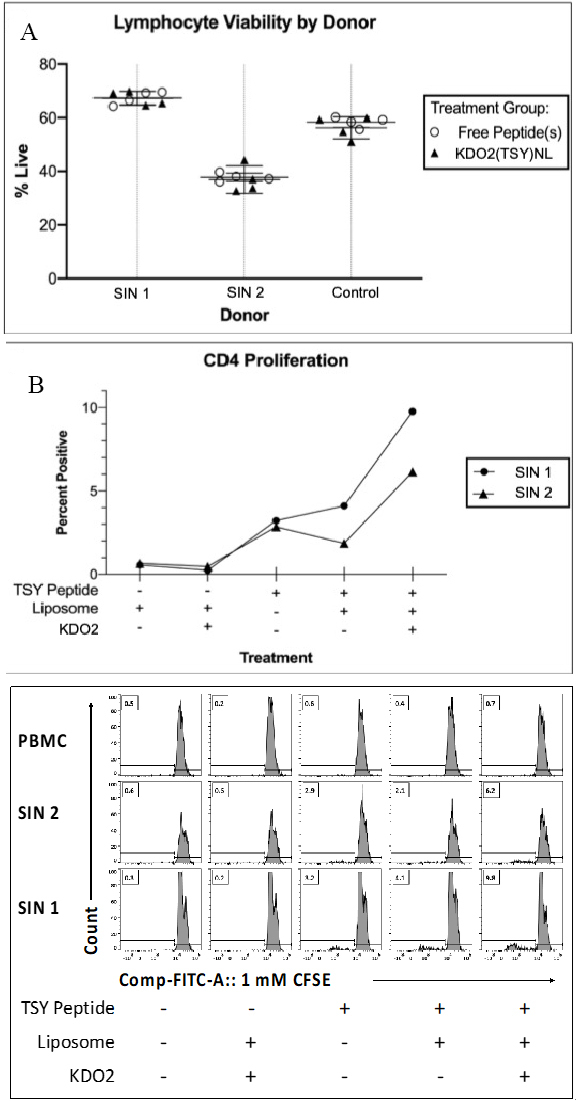
(A) Graph of lymphocyte viabilities of Sentinel Immunized Nodes (SIN) post-treatment. Thick horizontal bars represent mean viability among treatments for a donor; (B) showing a graph of average proliferation of CD4^+^ cell populations after culture treatments with (i) an empty anionic liposome, (ii) an anionic liposome containing KDO2 but no peptide; (iii) Free TSY (no liposome); (iv) TSY encapsulated in an anionic liposome; and (v) an anionic liposome containing TSY and KDO2; and (C) showing the 2D histograms used to calculate the data in 5B. PBMC: Peripheral blood mononuclear cells.

For all organs, the mice injected SQ displayed a higher concentration of fluorescent liposomes within the harvested organs than the IV-injected group. No overt signs of toxicity (lethargy, loss of appetite, weight loss) were observed in any of the mice over the study. Taken together, these data suggest that both IV and SQ administration of a KDO2-6MHP nanoliposome is safe, and that SQ administration of the KDO2-6MHP nanoliposome offers a robust delivery of cargo to critical secondary lymphoid organs (spleen and lymph nodes), which persists at least 6 days.

### urinActivation of CD4^+^ T cells from patients by a KDO2-MHP nanoliposome 

Using blood samples from melanoma patients who had previously been vaccinated with a 6MHP vaccine on the Mel41 trial and had developed a robust CD4^+^ T cell immune response to the TSY antigen^[39]^, we analyzed lymphocyte viability and CD4^+^ T cell proliferation to the TSY nano-formulations *ex vivo. *Under IRB guidance, we had previously collected fresh lymphocytes and sentinel immunized nodes (SIN) from these patients during that prior clinical trial. SIN were nodes draining the vaccine site, identified by lymphatic mapping techniques^[39,41]^ Using a live-dead marker and flow cytometry, we observed no discernable differences in cell viability between each of the patient samples re-treated with free non-encapsulated TSY peptide and the nanoliposomal KDO2/TSY formulation; however, the SIN sample from patient 2 (SIN2) had a decreased viability compared to SIN patient 1 (SIN1, Figure 5A). Viability was equivalent between a control group of patient samples that were not previously immunized with peptides [Figure 5A]. Since there was no apparent toxicity to incubation with nanoliposomes, we treated aliquots of each cell preparation with each of the following: (1) an empty ghost liposome; (2) a liposome containing KDO2 but no peptide; (3) Free TSY peptide (no liposome); and (4) TSY encapsulated within a liposome; and v) TSY within an immunogenic (KDO2 containing) liposome. Then the average proliferation of CD4^+^ gated cell populations for various culture treatment conditions was assessed [Figure 5B] by CFSE dilution over five days. Cell cultures were expanded from the harvested SIN biopsy of each donor, and CD4 T cell proliferation was assessed by CFSE dye dilution. Proliferation is reported as the percentage of CD3^+^ CD4^+^ gated population that have dividing (CFSE-diluted). Compared to all controls, both SIN1 and SIN2 donor cells responded to the combinational therapy. Figure 5C shows the 2D histograms of the same data against a negative control. Peripheral blood samples from each patient had no significant response to any of the treatments (data not shown). While an expected CD4^+^ T cell proliferation response is observed with free TSY and liposomal TSY, our data suggest an advantage of combining the peptide vaccine with a TLR4 agonist. It is worth noting that neither the liposome alone nor the KDO2-containing liposome alone triggers CD4^+^ proliferation, and thus KDO2 only acts to enhance the response to the peptide of interest. It should also be noted that while the SIN2 patient response is weaker than SIN1 [Figure 5B], this can be attributed to the slightly lower viability [Figure 5A] (and hence fewer live cells present) in the SIN2 donor cell population. Taken together, we demonstrate an immunogenic response to the KDO2/MHP nanoliposome *ex vivo*.

## DISCUSSION

In this study, we have developed nanoliposome formulations to encapsulate a specific panel of 6 melanoma helper peptides as a first step toward clinical application as a new melanoma vaccine strategy. The 6 peptides vary in length, hydrophobicity/hydrophilicity, and isoelectric point, which required creating 3 different nanoliposomal formulations to encompass them. These 6 peptides have previously shown modest immunogenicity and clinical activity as a vaccine when injected in their free form with other immunogenic adjuvants. Our goal was to improve future clinical efficacy through nano-enhancement strategies via simultaneous delivery of all 6MHPs and corresponding immunogenic adjuvants in a single nanoliposomal solution. In these investigations, we incorporated KDO2, a TLR4 agonist, into the lipid bilayer as our immune-stimulating adjuvant. Our results demonstrate that the individual liposomal formulations remain stable after being mixed together and that they can be simultaneously delivered without apparent toxicity *in vivo. *We show that via SQ injection, these 6MHP-loaded liposomes are capable of diffusing rapidly to secondary lymphoid organs and appear to remain in circulation for at least 6 days*.* We also show that these immunogenic liposome formulations significantly enhance immune responses to specific peptides *ex vivo*.

**Figure 6:**
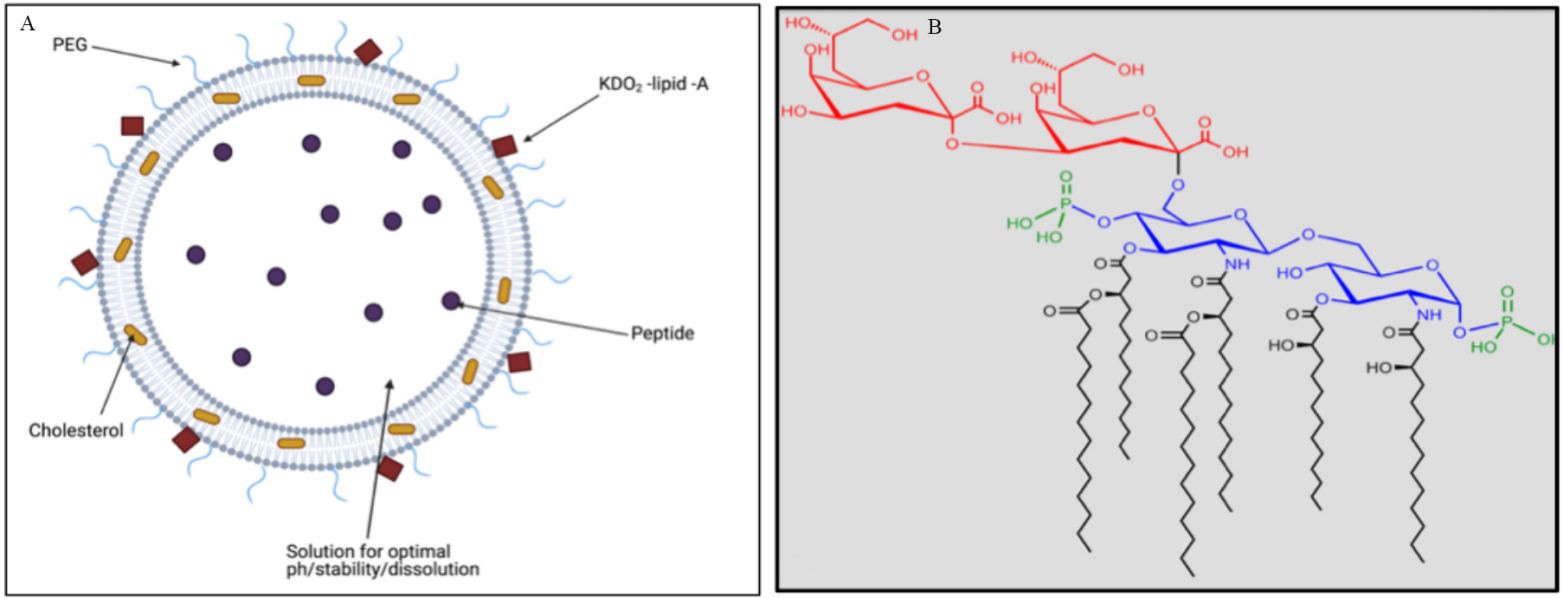
Showing (A) a schematic diagram of the unilamellar nanoliposome with the peptide dissolved within a stabilizing pH controlled buffer within the aqueous liposome core. Cholesterol embeds within the lipid bilayer; and a sparse PEG brush and immunogenic KDO2 lipid head group are arranged around the outer shell of the nanoliposome. The liposome is stored as a suspension in 1X PBS. It should be noted that additional lipid components are added to create either a positive or negative charge in some of the formulations. A fluorophore may also be added, which depending on the fluorophore used, may embed within the bilayer like cholesterol or be attached to a lipid and incorporate within the bilayer like PEG and KDO2. (B) shows the structure of KDO2-lipid A, which has 6 fatty acid chains and a head group on the surface.

The initial development of our nano-enhanced vaccine was complicated, as we were required to encapsulate and ultimately deliver 6 different peptides simultaneously. We selected a nanoliposome framework because of its versatility. Nanoliposomes can be made with a variety of different lipids to construct the lipid bilayer. They can be made to be neutral or charged, and the exterior surface can be modified chemically for the addition of targeting ligands that enhance liposome delivery; and/or stealth properties that increase biocompatibility and circulation time in the body^[42-44]^. Hydrophilic compounds are typically encapsulated within a nanoliposome’s aqueous interior, while hydrophobic (or lipophilic) compounds are typically embedded within the lipid bilayer. However, the development and optimization of nanoliposomes is not always a straightforward process, as overall charge, distribution of charge, and size, as well as ionic buffer strength, affect encapsulation efficiency. Each of our peptides had different physical properties, so a single liposome formulation was not optimal for this range of peptides. Instead, we engineered 3 formulations and encapsulated each peptide based upon pH-dependent solubility. While each formulation may have a different surface charge or be formulated with a different buffer system, they still all include a fixed PC/PE ratio that maintains stability, a fixed cholesterol content that prevents leakiness, a fluoroprobe to facilitate *in vivo* imaging, a reduced PEG brush to help trigger the body’s T cell response to these liposomes, and a low concentration of the TLR4-agonist, KDO2, to enhance the adaptive immune response without inducing systemic toxicities. Figure 6A shows a schematic diagram of our base nanoliposome formulation, and Figure 6B shows the chemical structure of KDO2-Lipid A.

The new formulations and pH-controlled buffers improved the encapsulation efficiency of most of the peptides compared to the use of a generic neutral nanoliposome formulation. TSY was improved the most. TSY is most stable in pH 5-5.5; thus, PBS did not provide the ideal buffer conditions, while the MES buffer used in our optimized formulations was much more favorable. The anionic liposome formulation also aided in improving the encapsulation of the positively-charged TSY peptide. Similarly, AQN, which had very poor encapsulation in our neutral formulation, also achieved huge encapsulation enhancements when dissolved in a buffer that maintained an alkaline pH and by using a cationic charged nanoliposome to further enhance the encapsulation of the negatively charged peptide. By contrast, encapsulation of WNR, which has a slightly positive charge, was only marginally improved after switching to an anionic liposome and buffer that maintained a pH of 7.0-8.0. LLK, FLL, and RNG achieved reasonable encapsulation values in our neutral formulation, so we did not expect significant improvements with a switch of buffers. However, for LLK and FLL, but not RNG, dissolving the peptides in LR/NaHCO_3_ buffer, instead of PBS, slightly improved encapsulation efficiencies. Based on previous clinical studies, where 200 ug of each peptide was delivered to human patients^[39]^, we estimate that a liposomal loading value of ~50ug/ml should yield an effective immunologic dose. Our studies show that LLK, FLL, and TSY can be reengineered to reach and/or exceed this therapeutic dose. For AQN, WNR, and RNG, we may also attempt pH-dependent active methods of liposomal loading to further enhance encapsulation of these peptides within these nanoformulations. However, based upon previous studies from our group^[22]^, we might expect that the nanoformulations, in fact, better protect and deliver the peptides, allowing suboptimal encapsulation efficiencies to now reach target therapeutic doses.

In future studies, to further improve liposomal MHP encapsulation, we may also attempt to modify the peptides via techniques such as myristoylation^[45]^ or palmitoylation^[46]^. Both of these methods have been shown to enhance peptide-lipid interactions, allowing for intercalation of the peptides within the lipid bilayer. We chose not to adopt this process initially for several reasons. First, as we already intercalated cholesterol, KDO2, and our fluorophore within the lipid bilayer, we wanted our peptides to sit within the aqueous core of the liposomes in their native form. We were also concerned that incorporation within the lipid bilayer might lower the efficacy of our therapy due to prolonged release kinetics and/or destructive interaction/competition with the other adjuvants already in the lipid bilayer. We were also uncertain about how such modifications (and changes in peptide configuration) might affect the immunogenicity of the parent MHP. As part of this ongoing work, we plan to test whether these (and similar) modifications preserve or alter the immunogenicity of the 6MHPs.

A major innovation of our nano-encapsulated approach is the incorporation of the immunogenic KDO2-lipid A into our core nano-liposome formulation. The present study demonstrates the feasibility of creating customized nanoliposomes, containing varying peptides, which can then be mixed for storage and co-administration. These data provide a platform on which to build even more promising nanoliposome strategies. One we propose is to add an antibody that can target the adjuvanted nanoliposomes specifically to dendritic cells. In murine studies, very strong circulating T cell responses, representing about 50% of circulating T cells, have been induced by IV vaccination with peptides plus a TLR agonist and a CD40 antibody that targets antigen-presenting cells (TriVax)^[47-50]^. However, comparable systemic dosing of those agents for humans is not likely achievable without unacceptable toxicity. If, on the other hand, these 3 agents could be co-administered in a nanoparticle targeted to dendritic cells (DC), it may prove as effective with a much lower dose. Future versions of our immunogenic liposomes will include bioconjugation strategies (EDC/NHS) for binding CD40 Ab to the surface of the liposomes. 

Our biodistribution studies, conducted in healthy murine subjects, suggest that SQ administration could be a preferred method of delivery for our nano-vaccines. Systemic (IV) delivery seems to rapidly distribute the liposomes non-specifically throughout the body immediately after injection, and also appears to then produce a more rapid clearing of them from the body. On the other hand, SQ delivery allows for a slower and more controlled distribution. We acknowledge that a limitation of this present work includes not knowing precisely the extent to which the fluorescent tracer may be released over time *in vivo.* However, as the fluorophore is conjugated to a lipid within the bilayer, we do not expect significant amounts of tracer to be released until the liposomes themselves begin breaking down. Our benchtop studies at body temperature in 10% bovine serum showed that while we observed peptide release after 24 h, the liposomes themselves remained stable for several days. We are confident that the fluorescent distributions imaged immediately after injection on day zero [Figure 3A and C] are representative of liposome distribution at that time. Unlike IV delivery, SQ delivery does not immediately distribute systemically but does show the rapid appearance of the liposomes within the lymph nodes and spleen. This is encouraging and implies that the nanoliposomes exhibit immunogenicity. It also suggests that even without specific dendritic cell targeting, the nanoliposomes distribute in tissues with high concentrations of dendritic cells, which can be expected to support T cell activation. In future studies, we intend to conduct more detailed benchtop tests to specifically monitor fluoroprobe release over time, as well as increase the frequency of live animal imaging. We also plan to extend this work into a B16 melanoma mouse model. This will require exchanging our humanized antigens with mouse antigens, which may require some alterations to the nanoformulations and thus was beyond the scope of this manuscript. However, this kind of study will allow us to evaluate biodistribution, pharmacokinetics (PK), and immunogenicity of the KDO2-nanoliposomes, as well as determination of pharmacodynamics (PD) for the efficacy of our nanoformulations in a mouse model. We would also like to repeat studies in mice with depletion of dendritic cells to confirm the reliance on those cells.

The goal of the present work was to test whether the 6MHPs could be encapsulated into nanoliposomes with a TLR agonist and whether these would be stable and show preliminary evidence of enhanced immunogenicity. In our preliminary investigations, the TSY peptide consistently achieved the highest liposomal encapsulation values and was also strongly immunogenic in a melanoma patient population^[41]^. Our team also had access to 2 patient samples, previously documented to exhibit a strong TSY immunogenic response. Thus, as a proof-of-concept, we evaluated proliferative responses of human CD4^+^ T cells to the TSY peptide *ex vivo *using these 2 patient samples. Our assays showed an enhanced immunogenic response with the peptide encapsulated within a KDO2-nanoliposome, compared to formulations without KDO2 and the free peptide. We expect that this enhancement is mediated by dendritic cell activation and subsequent helper peptide presentations to melanoma-reactive T cells. These results are in line with clinical data demonstrating that simultaneous delivery of a TLR4 agonist with immunogenic peptides enhances T-cell activation^[16]^. These initial studies provide a basis for carrying forward this study, first by performing *ex vivo* activation of the five remaining antigens individually, as well as *ex vivo *analysis of the “full vaccine” with a mixture of all 6 peptide nanoliposomes.

Future experiments to support the preclinical development of our nano-formulations include formal PK and toxicological studies by a contract research organization, in addition to the determination of PD for efficacy in a mouse model.

Our results show promise in the use of custom-designed immunogenic (KDO2) nanoliposomes as the delivery vehicle for cancer vaccines. Co-delivery of antigens plus the TLR agonist KDO2 to antigen-presenting cells offer promise to enhance immune responses to melanoma antigens. Prior vaccines in humans commonly induce weak or transient T cell responses: by enhancing those immune responses, this new strategy offers promise to overcome weak antitumor immunity and enable immune-mediated control of melanoma. Moreover, by improving the delivery and efficacy of nano-cancer vaccines, drug resistance to current chemotherapies can also be overcome. Our mouse studies show that a subcutaneous injection may have advantages over IV injection, as it allows the nanoliposome vaccine to concentrate and persist within tissues and organs with high DC populations. This will provide an easier route to immunization that is cost-effective and less invasive for the patients receiving care. Co-administering diverse adjuvants within a nanoliposome are expected to show even further enhanced responses *in vivo*, as it provides a way of targeted delivery, ensuring that all adjuvants are delivered directly and simultaneously to DCs.

## DECLARATIONS

### Copyright

© The Author(s) 2022.
